# Nicotine reduces discrimination between threat and safety in the hippocampus, nucleus accumbens and amygdala

**DOI:** 10.1038/s41398-024-03040-5

**Published:** 2024-08-03

**Authors:** Madeleine Mueller, Tahmine Fadai, Jonas Rauh, Jan Haaker

**Affiliations:** 1 University Medical Center Hamburg-Eppendorf (Germany), Department of Systems Neuroscience, Hamburg, Germany; 2 University Medical Center Hamburg-Eppendorf (Germany), Department of Child- and Adolescent Psychiatry and Psychotherapy, Hamburg, Germany; 3 University Medical Center Hamburg-Eppendorf (Germany), Department of Psychiatry and Psychotherapy, Psychiatry Neuroimaging Branch, Hamburg, Germany

**Keywords:** Hippocampus, Human behaviour

## Abstract

Nicotine intake is linked to the maintenance and development of anxiety disorders and impairs adaptive discrimination of threat and safety in rodents and humans. Yet, it is unclear if nicotine exerts a causal pharmacological effect on the affective and neural mechanisms that underlie aversive learning. We conducted a pre-registered, pseudo-randomly and double-blinded pharmacological fMRI study to investigate the effect of acute nicotine on Fear Acquisition and Extinction in non-smokers (n = 88). Our results show that nicotine administration led to decreased discrimination between threat and safety in subjective fear. Nicotine furthermore decreased differential (threat vs. safety) activation in the hippocampus, which was functionally coupled with Nucleus Accumbens and amygdala, compared to placebo controls. Additionally, nicotine led to enhanced physiological arousal to learned threats and overactivation of the ventral tegmental area. This study provides mechanistic evidence that single doses of nicotine impair neural substrates of adaptive aversive learning in line with the risk for the development of pathological anxiety.

## Introduction

A key mechanism for coping with a threatening environment is aversive learning [1]. Adaptive aversive learning enables individuals to discriminate between what is dangerous and what is safe in their surroundings [2]. Maladaptive learning is characterized by impaired discrimination between threats and safety, for example evident in individuals that are suffering from anxiety disorders (AD) [3]. Patients diagnosed with AD have higher smoking rates, compared to healthy individuals [4] and it is possible that smoking is associated with maladaptive aversive learning, as healthy smokers show impaired safety learning, compared to healthy non-smokers [5]. Moreover, a prospective longitudinal study indicated that smoking in healthy individuals results in a higher risk for the onset of a panic attack [6], suggesting that smoking might contribute to maladaptive learning. But the key question is how nicotine, the psychoactive ingredient in cigarette smoke, causally interferes with aversive learning, leading to impaired discrimination between safety and danger.

One possibility is that nicotine affects the neural systems that are engaged by aversive learning and impacts on synaptic plasticity in these neural regions. In animals, there is evidence that nicotine affects synaptic changes that underlie learning and associative memory formation. These effects could be mediated by neural activation on the systems level, since infusion of nicotine changes neural activity in brain regions that are key for emotional learning, such as the amygdala, hippocampus and medial prefrontal cortex (mPFC) [7]. On the cellular level, nicotine has a direct influence on synaptic plasticity in the hippocampus, where it binds to α4*β*2-containing nicotinic acetylcholine receptors (nAChR) [8] and modulates different memory-related processes including short-term potentiation (STP) and long-term potentiation (LTP) [9, 10]. The hippocampus is important to process new and salient information about threats and stores memories that result from aversive learning [11]. Such aversive learning is commonly examined in laboratory protocols of fear conditioning. During Fear Acquisition (ACQ) a conditioned stimulus (CS+) is learned as a danger signal, based on the prediction for an aversive, unconditioned stimulus (US, e.g. an electric stimulus). Another conditioned stimulus (CS-) is learned as a safety signal and predictive for the absence of the US. Indicative of adaptive learning, subjects learn to discriminate between CS+ and CS-, reflected by differential (i.e., higher for the CS+ as compared to the CS-) conditioned responses [12].

Chronic nicotine intake, such as smoking, seems to impair adaptive discrimination of explicit identification of threat and safety, but with enhanced autonomic arousal (skin conductance respones (SCR) [13]) in fear conditioning protocols. As such, a chronic schedule of nicotine administration in mice, and smoking in humans, has shown to decrease the discrimination between CS+ and CS-^14^, in explicit rating in humans, and defensive responses in rodents. Similarly, experiments in rodents revealed that the discrimination between threat and safety (contexts) is dose-dependently impaired by acute nicotine administration [15]. Supporting this finding, acute infusion of nicotine into the dorsal hippocampus (HC) disrupted safety learning in rodents [16]. These findings are related to results in humans, showing that smokers have an impaired discrimination between threat and safe contexts, when compared to non-smokers, indicated by lower discrimination in subjective fear, US expectancy and SCR [17]. A similar impairment in smokers was found in another study, which revealed less discrimination in self-reported fear towards learned threat and safety cues, compared to non-smoking individuals [5].

While smoking seems to impair discrimination of threat and safety, it is unclear if there is a causal pharmacological mechanism by which nicotine affects the discrimination of threat and safety. Such a pharmacological effect could aid to explain maladaptive learning that is observed in smokers. The preregistered main hypothesis of this study is that acute nicotine reduces discrimination between threat and safety during aversive learning and extinction training ((CS+_placebo_ > CS-_placebo_)>(CS+_nicotine_ > CS-_nicotine_); see https://drks.de/search/en/trial/DRKS00025233) in humans. This was expected for all our outcome measures, i.e. subjective ratings, skin conductance responses and neural activity in our regions of interest.

## Methods

### Participants

According to power analysis (see supplement), 88 healthy, non-smokers between 18 and 40 years were recruited for this study. Individuals confirmed to have no neuropsychiatric diagnoses, to consume less than 15 units of alcohol/week, to have not taken any prescribed medication in the two months prior to study entry, and to have not used any illicit (e.g. psychotropic) drugs in their lifetime (Table 1). Subjects had to be suitable for MRI measurements. “Non-smoker” was defined by not being an active smoker during the time of the data acquisition and having smoked <200 cigarettes during lifetime. Two participants had to be excluded from the analysis due to false statements and one participant was excluded due to too high alcohol consumption resulting in a final sample of 85 subjects (55.8% female). Three participants only completed day 1 of the study and are therefore excluded from day 2 analyses. All participants gave written, informed consent to participate and received 120 C reimbursement. The study was approved by the local ethics committee (Ethikkommission der Ärztekammer Hamburg, PV5514) and complies with the Declaration of Helsinki.

**Table 1:** Demographics per group.

	Nic1 Mean (sd)	Nic2 Mean (sd)	Pla Mean (sd)	Group statistic
Sample size	30	29	26	
Gender	56.67% female	55.17% female	55.56% female	p = 0.982^2^
Age [years]	24.79 (4.39)	26.17 (5.0)	25.35 (3.98)	p = 0.504^1^
Coffee consumption [cups/day]	0.89 (0.97)	0.98 (1.22)	0.7 (0.77)	p = 0.588^1^
Alcohol consumption [drinks/week]	1.62 (2.07)	0.83 (1.17)	1.43 (1.43)	p = 0.161^1^
STAI-T	31.33 (6.23)	32.59 (6.72)	34.69 (7.49)	p = 0.186^1^
STAI-S [day 1]	44.23 (2.64)	44.79 (1.8)	44.58 (2.45)	p = 0.648^1^
STAI-S [day 2]	45.5 (2.32)	44.107 (2.35)	45.08 (1.98)	p = 0.059^1^
Attention test - Day 1 [concentration performance score]	252.43 (46.06)	257.68 (38.88)	266.74 (38.31)	p = 0.439^1^
Attention test - Day 2 [concentration performance score]	272.03 (31.38)	263.25 (37.89)	273.24 (33.26)	p = 0.496^1^
Body Mass Index	23.25 (2.70)	23.67 (3.70)	22.92 (3.17)	p = 0.807^1^
Side effects day 1 [0–6]	1.34 (0.9)	1.34 (0.93)	1.19 (0.61)	p = 0.351^1^
Side effects day 2 [0–6]	1.23 (0.74)	1.33 (0.82)	1.22 (0.72)	p = 0.602^1^
Guessed correctly, when asked to which group assigned	56.67%	44.83%	38.46%	p = 0.058^2^
Contingency (correct identified CS+ post experiment)	94.74%	95.24%	95.0%	p = 0.780^2^

Nic1 received nicotine before Fear Acquisition, Nic2 received nicotine before Extinction training and the control group received placebo before both days. Group statistics were calculated using ANOVA with each demographic score as the dependent variable and group as the fixed factor (marked as^1^) or using a Chi-Square test of independence with contingency tables for binomial data (marked as^2^).

### Groups

To test the effect of acute nicotine on aversive learning in a Fear Acquisition and Extinction training (EXT) protocol including a reinstatement test, we compared three groups. Group 1 (Nic1) received 1 mg nicotine before ACQ on day 1 and then placebo before EXT on day 2. Group 2 (Nic2) received a placebo before ACQ on day 1 and then 1 mg nicotine before the EXT on day 2. Group 3 (Pla) as the control group received placebo before both days. The assignment to each group was randomized and the administration of the drug was double-blinded. The three groups did not differ in demographic parameters that were assessed in this study (Table 1).

### Stimulus material

We used a two day context-dependent cue conditioning paradigm, which is an adaptation of an already publicized protocol [18]. The context was a virtual room that was presented on a screen (Source Engine,Valve Corporation,Bellevue,USA [19]). Three different contexts were used, where context A and context B were both virtual offices but differed in their set-up and context C was a mixture between context A and context B. Each context was presented from two different viewpoints. The virtual rooms could be illuminated by either yellow or blue coloured light, which served as cues for fear conditioning [18]. One cue (CS + ,duration=6 s) was predictive for an aversive electric stimulus (US;5.5 s after CS+ onset), whereas another cue (CS-,duration=6 s) was not reinforced. Colours of the cues were counterbalanced. Context-presentations without cues were used as inter-trial intervals (ITIs, duration range between 7 and 11 s). Visual stimuli were presented using Presentation® software (Version20.3,Neurobehavioral Systems,Inc.,Berkeley,CA,www.neurobs.com). The electrotactile stimulus that served as a US consisted of a train of 3 pulses, each with a duration of 2 ms and an interval of 50 ms. The US was delivered via a surface electrode (Specialty Developments,Bexley,UK) on the dorsal surface of the right hand using a DS7A electrical stimulator (Digitimer,Welwyn Garden City,UK). The US intensity was individually adjusted prior to ACQ to a threshold that was perceived as very unpleasant, but not hurtful (mean=2.28 mA,sd=4.09,min=0.4 mA,max=37 mA; no difference between groups).

### Experimental procedure

**Fig. 1:**
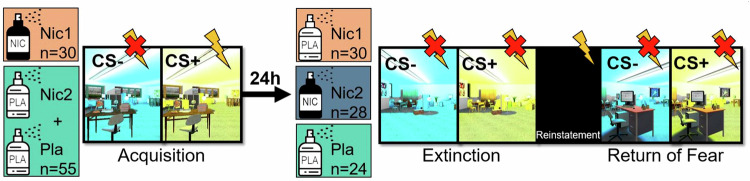
Experimental procedure. The group Nic1 received nicotine before the fear acquisition on day 1 (ACQ), all remaining subjects received placebo. After 24 h the group Nic2 received nicotine and the remaining subjects received Placebo. Following that, subjects performed the Extinction training (EXT) with a subsequent Return of Fear (RoF) manipulation in form of a Reinstatement. CS colours were counterbalanced.

The experimental paradigm (Fig. 1) consisted of a Fear Acquisition (ACQ) on the first day, followed approximately 24 h later by an Extinction training (EXT) with a Return of Fear manipulation (RoF) in form of a reinstatement. Both experimental days were performed in the fMRI scanner. Depending on the pseudo-randomly assigned group, either 1 mg nicotine or 1 mg placebo was administered double-blinded 15minbefore participants started the experiment (to reach the plasma maximum). During ACQ, 4 trials of stimulus habituation without reinforcement were followed by 3 blocks with each 8 CS+ (reinforcement rate=75%) and 8 CS- presentations. The EXT consisted of two blocks with each 8 CS+ and 8 CS- presentations (reinforcement rate=0%). Subsequently, the reinstatement took place (4 US were delivered companying a black screen). That was followed by a reinstatement test (one block with 8 CS+ and 8 CS- presentations; reinforcement rate=0%). Return of Fear results can be found in the Supplement. During ACQ, participants saw context A and during EXT participants saw context B. In the reinstatement test, participants saw context C. This study was preregistered at the German Clinical Trials Register (Deutsches Register Klinischer Studien (DRKS); DRKSID: DRKS00025233).

### Pharmacological intervention

Participants received 1 mg nicotine as oral-spray (Nicorette®-Spray, Johnson&Johnson GmbH) and 1 mg placebo as oral-spray (St. Severin Cayenne-Pepper-Spray®, HECHT Pharma GmbH,27432 Bremervörde), or placebo on both days. The dose was tested in a prior pilot study and proofed to be effective with little side effects. Typical and self-reported side effects were documented after both experimental days. Participants rated their perception on a scale from 0 (no side effect) to 6 (extreme side effect). No difference of side effects was found between participants receiving nicotine and participants receiving placebo (mean_Nic_ = 0.34,sd_Nic_ = 0.86;mean_Pla_ = 0.25,sd_Pla_ = 0.76).

### Outcome measures

#### Ratings

Participants rated how much fear/stress they felt towards the CSs and the ITIs on a visual Analogue Scale [VAS,0(none)–100(maximally)] before and after ACQ as well as before and after EXT and after the reinstatement test. Preceding the US presentation, subjects rated their US expectancy as a binary choice (yes/no) trialwise on both experimental days.

#### SCR

Skin conductance responses (SCR) were measured on the hypothenar region of the left hand of the participant. Raw data was scored manually (see Supplement). Scored data was normalized for each day and participant.

#### Regions of interest

Regions of interest (ROI) were defined as key structures in emotional processing and fear, such as the bilateral amygdala, hippocampus, insular cortex, dorsal anterior cingulate cortex (dACC) and ventromedial prefrontal cortex (vmPFC), and dopaminergic innervated key structures, such as the Nucleus Accumbens (NAcc) and the ventral tegmental area (SN/VTA). The amygdala, the hippocampus, the insular cortex and the NAcc were defined by Harvard-Oxford probability maps. For the dACC and vmPFC were no anatomical masks available, therefore we defined these two ROIs as in previous studies [20, 21]. The vmPFC ROI was defined as a box of 20 × 16 × 16 mm at x = 0 y = 42 z = -12. The dACC ROI was defined as a box of 20 × 16 × 16 mm at x = 0 y = 28 z = 26. The SN/VTA complex was defined by Bunzeck et al. [22]. Correction for multiple comparisons within these ROIs was performed by using family-wise error correction based on the Gaussian Random Fields as implemented in SPM.

#### Questionnaires

Participants gave information on age, gender, alcohol and coffee consumption, as well as their smoking behaviour. Furthermore, participants completed the State-Trait Anxiety Inventory (STAI-S/STAI-T) [23]. To check if nicotine has a general effect on attention, participants completed the d2-Test of attention after each experimental day [24] (see Supplement).

### Data analysis

#### Rating/SCR

The analyses of the fear ratings (pre/post), the US expectancy (trialwise), as well as the SCR (trialwise) were performed using linear mixed effect models in R with the lme4 package [25]. As preregistered, we focused the analysis on the last block/post ratings of the ACQ and EXT. The dependent variable in the models were the ratings or SCR, respectively. Additionally, we implemented random intercepts for subjects. To test the paradigm, we analysed the interaction between stimuli types (CS + /CS-) in the post fear ratings and during the last block for US expectancy/SCR and the groups (ACQ: nicotine (Nic1)/placebo (Nic2 and Pla); EXT: nicotine before ACQ(Nic1)/nicotine before EXT(Nic2)/placebo(Pla)): (RatingResults/SCR∼(1|participants)+stimulus*group). To further investigate group effects across outcome measures, we used the same model, but the stimulus discrimination (CS + -CS-) was calculated as dependent variable: StimulusDifference∼(1 |participants)+group. To test the results estimated marginal means (EMMs) were computed using the emmeans package as post-hoc tests and p-values were corrected for multiple comparisons using Bonferroni-Holms method. Regarding the differential post ACQ fear ratings, we used jasp [26] to calculate an ANOVA with the dependent variable of the stimulus difference and calculated follow up post-hoc tests.

#### fMRI

Preprocessing and statistical analysis of functional MRI data was carried out using SPM12 (Statistical Parametric Mapping,http://www.fil.ion.ucl.ac.uk/spm) running under MATLAB R2021b (The MathWorks,Inc.,Natick,Massachusetts,US). Before preprocessing, the first five volumes of each time series were discarded to account for T1 equilibrium effects. Remaining images were unwarped, realigned to the first image, coregistered to the individual high resolution T1 structural image, normalized (using DARTEL) and smoothed. Following statistical analyses were performed using a general linear model (GLM) at the single-subject level as standard approach for fMRI implemented in the SPM software. Experimental conditions (i.e., CS + , CS-, (omitted) US, introductions, ratings, button presses) were defined as separate regressors (stick function) modelling the predicted time courses of experimentally induced brain activation changes. Subsequently a full-factorial analysis was calculated on the group level. As preregistered, we focused our analyses on the last block of ACQ and EXT, but added analyses of the other blocks. Additional analyses on both days were performed in which the US expectancy of each participant was used as parametric modulator (i.e., expectation of no US>expectation of a US). Participants that showed minimal variation in rated US expectancy (i.e., only one trial with no US expectancy) where removed from the analysis.

#### Connectivity analyses

To investigate functional connectivity differences in stimulus discrimination between groups, we employed psycho-physiological interactions (PPI,SPM12 standard approach) for the last block of the ACQ and EXT. As seed region served the left hippocampus as described in our ROIs. We then used the PPIs of each participant as regressor in an individual GLM, including movement as regressor. Finally, calculated estimates we contrasted on group level.

## Results

### Acquisition

Participants discriminated between CS+ and CS- at the end of the Acquisition (ACQ) (last block/post Rating;pre-registered contrast) in fear ratings, US expectancy and SCR across all groups (Table S1/Fig. S1–S3).

#### Group effects

In order to test if acute nicotine administration reduces CS-discrimination at the end of Fear Acquisition training, we compared CS-responses between the group that received nicotine (Nic1) with individuals that received placebo on day 1 (Pla). Our preregistered analysed focused on the last block during acquisition training (8 trials of each CS), based on our pilot data on US expectancy. We found no gender differences in the results relating to the pharmacological groups (see Supplement).

##### Fear rating

**Fig. 2:**
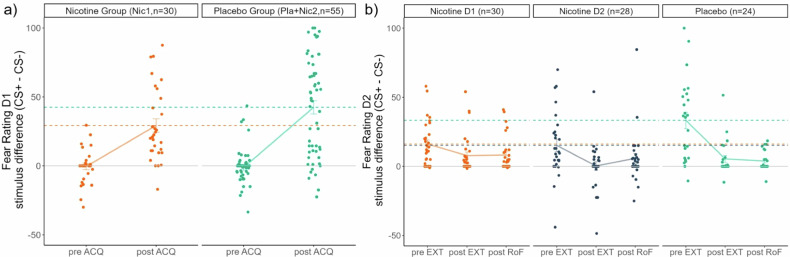
Fear ratings. Weaker stimulus discrimination in the groups that received nicotine when compared to the Placebo group in fear ratings, (**a**) after Fear Acquisition (day 1), (**b**) before Extinction (day 2). No group differences in the differential fear ratings were found pre ACQ, post EXT or post RoF. Single subject responses are shown as scatterpoints, mean differential fear ratings are depicted as lines with standard errors. Dashed lines represent the mean differential fear ratings per group post ACQ/pre EXT.

According to our hypothesis, we found lower differential fear ratings (CS + > CS-) after the ACQ in the group that received nicotine, when compared to the placebo group (preregistered post-hoc results:(t(165) = 2.296,p_corr_ = 0.046); ANOVA:time*group interaction (F(1,83) = 3.155,p = 0.079); Fig. 2a). This effect was driven by lower fear ratings towards the CS+ in the group that received nicotine, compared to the placebo group (t(83) = 2.00,p = 0.049; Fig. 2a). There was no group effect in the analysis of the fear ratings before ACQ (F(1,83) = 0.043,p = 0.836), indicating that nicotine did not change baseline levels of subjective fear.

##### US expectancy

We found no group effect on US expectancy in the last block of the ACQ (F(1,83) = 0.032, p = 0.859; Fig. S1).

##### SCR

We found a stimulus*group interaction (F(1,928) = 6.182,p = 0.013, Fig. S3), with higher differential skin conductance response in the group that received nicotine, when compared to the placebo group (t(60.1) = 2.292,p_corr_ = 0.025; Fig. S3). This enhancement in differentiation by nicotine was contrary to our pre-registered hypothesis and comparison of CSspecific responses between groups revealed no differences.

##### fMRI

**Fig. 3:**
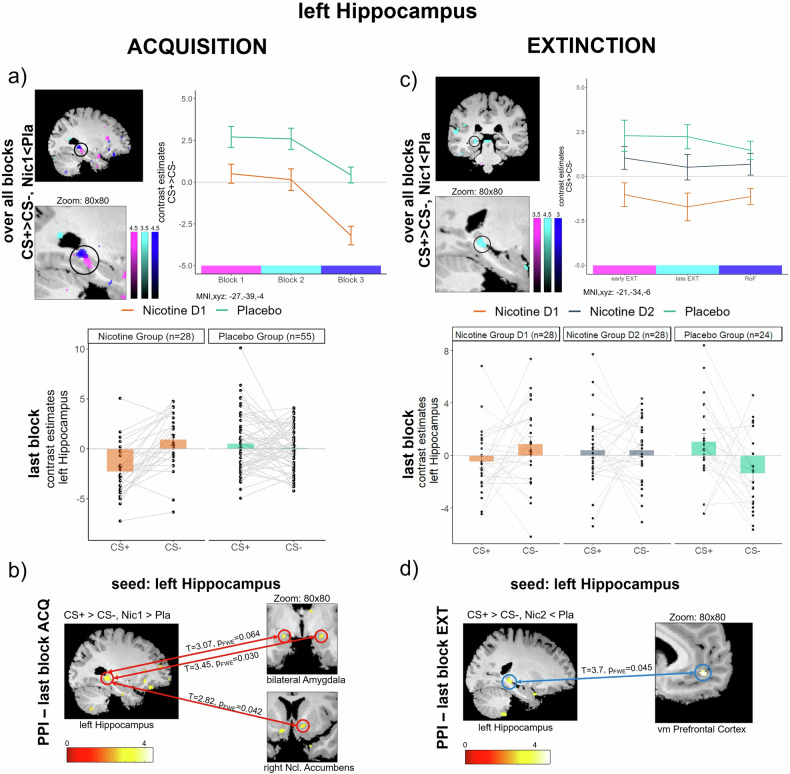
Hippocampal activity. **a** Acquisition: Acute nicotine administration, compared to placebo, reduces differential responses to the CS+ and the CS- during the last block of ACQ in the left hippocampus (similar to fear rating results). This effect is robust over all three blocks of the ACQ in the left HC. **b** Psycho-physiological interaction (PPI) during the last block of ACQ with left hippocampus as seed region. Nicotine administration before ACQ leads to increased connectivity towards the bilateral AMY and the right NAcc, compared to placebo controls. **c** Extinction: Weaker stimulus discrimination during late EXT in the left HC in the group that received nicotine before ACQ (Nic1), when compared to placebo controls. No group differences in hippocampal activity during EXT or RoF were found between Nic2 and Pla. Scatterpoints represent single subject parameter estimates to each CS. Bars represent means across each group with standard error. **d** Psycho-physiological interaction (PPI) during the last block of EXT with left hippocampus as seed region. Nicotine administration before EXT leads to decreased connectivity towards the vmPFC, compared to placebo controls.

**Fig. 4:**
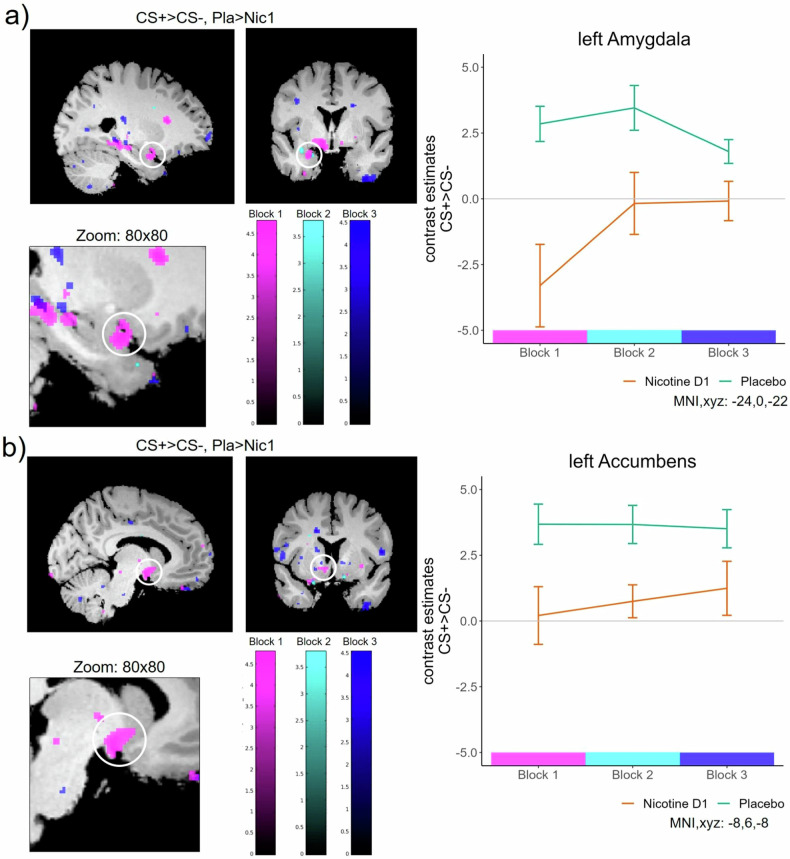
Stimulus discrimination during Fear Acquisition. fMRI results over all blocks in the (**a**) left Amygdala and (**b**) left Ncl. Accumbens. The group that received nicotine (Nic1) shows a lower activity in the ROIs over time, when compared to the Placebo group (contrast: CS + > CS-, Pla>Nic1).

First, we aimed to delineate the neural effect of reduced CS-differentiation by nicotine which parallels the reduced CS-differentiation in subjective fear. This analysis revealed reduced differential (CS + > CS-) responses in the left (and trendwise in the right) hippocampus in the group that received nicotine, compared to the Placebo group (left HC: T = 3.77,p_FWE_ = 0.016;right HC:T = 3.36,p_FWE_ = 0.055; Table S1/Fig. 3a). This effect was driven by a reduction in responses to the CS+ and thereby mirrored the reduced discrimination in rated fear in the group that received nicotine, compared to Placebo controls. The inverse contrast (i.e., enhanced discriminatory responses in the Nicotine, vs. the Placebo group) revealed no voxel within our ROIs. In order to establish the effect of nicotine on HC activation within the earlier stages of aversive learning, we compared the differential responses (CS + > CS-) between groups in the first and second block of ACQ, as an exploratory analysis. Similar, to the last block of ACQ, we found a decrease in the differential activation of the bilateral HC (left HC:T = 3.60,p_FWE_ = 0.026;right HC:T = 3.53,p_FWE_ = 0.033) in the group that received nicotine, compared to the placebo group in the first block (Table S1). In addition to this effect in the HC, we found decreased differential responses in the left AMY in the group that received nicotine, compared to the placebo group in the first block and trendwise in the second block of the ACQ (first block;left AMY: T = 3.68,p_FWE_ = 0.009; second block; left AMY:T = 3.03,p_FWE_ = 0.054;Table S1). We also found a similar effect in the left NAcc in the first block (T = 3.03,p_FWE_ = 0.023). The adaptive differentiation between CS+ and CS- during aversive learning is reflected in the hippocampal (Fig. 3a), amygdala and NAcc (Fig. 4) responses within the Placebo group, but is impaired after administration of nicotine.

##### Parametric modulations

**Fig. 5:**
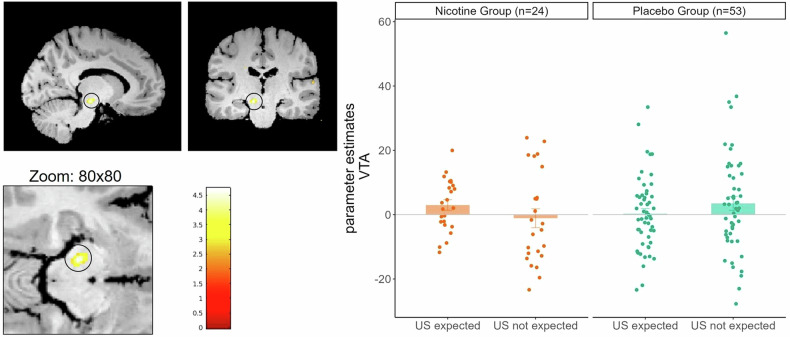
Parametric Modulation of US expectancy in SN/VTA. Stronger activation in the SN/VTA in the Nicotine group, when subjects expected the US, when compared to the placebo group over the whole fear acquisition. Scatterpoints represent single subject contrast estimates in the SN/VTA, bars represent means with standard errors.

In order to follow the individual learning of threat contingencies, we entered individual US expectancy ratings (US expected:1, US not expected:-1) as a modulator for the activity towards the CS + . Hence, the activation in this analysis would reflect responses in a brain region that is modulated by US expectation during CS+ presentation (this analysis was similar to the preregistered analysis for the EXT, including the whole ACQ time-course). Here, we found that the activity in SN/VTA was modulated by US expectancy during CS+ presentation, which was altered by nicotine administration (MNIxyz:-10,-21,-12;T = 4.80,p_FWE_ < 0.001; Fig. 5). Specifically, we found activation in the placebo group in the SN/VTA when the US was not expected (Fig. 5 most right barplot). In contrast, nicotine administration led to activation of the SN/VTA when the US was expected (Fig. 5 most left barplot). In other words, the activity in the Placebo group decreased with increasing expectancy in the SN/VTA, whereas nicotine administration led to constant responses when participants expected a US.

##### PPI

To further investigate the linkage of the nicotine effect in the hippocampus and other ROIs, we employed a functional connectivity analysis in form of a psycho-physiological interaction (PPI). Our PPI analyses with the left hippocampus as a seed region was calculated for the last block of ACQ. We found increased functional connectivity in the group that received nicotine before ACQ between the left hippocampus and the bilateral AMY (left AMY:MNIxyz:-20,-6,-15;T = 3.07,p_FWE_ = 0.064;right AMY:MNIxyz:27,0,-14;T = 3.45,p_FWE_ = 0.030) as well as the right Ncl. Accumbens (MNIxyz:12,9,-10;T = 2.82;p_FWE_ = 0.042), when compared to the Placebo group (Fig. 3b).

### Extinction

On day 2, participants underwent Extinction training (EXT), in which CS-discrimination was still evident at the end of EXT across groups, indicated by higher fear ratings, US expectancy, trendwise higher SCRs and increased neural activity in multiple ROIs (Table S2/Fig. S1–S3).

#### Group effects

In order to examine if prior nicotine administration during acquisition training (Nic1), as well as acute nicotine administration during extinction (Nic2) resulted in diminished CS-discrimination, as compared to placebo (Pla), we compared responses between all three groups (Nic1/Nic2/Pla) in the last EXT block (i.e., eight trials; see preregistration).

##### Fear rating

Against our hypotheses, we found no effect of nicotine on the differential fear ratings at the end of EXT. Our secondary analyses compared CS-discrimination in subjective fear that was given before EXT, reflecting the retrieval of learned threat memories from day 1. Before EXT, we found a stimulus*group interaction (F(2,79) = 4.949,p = 0.009) with reduced differential fear rating in both nicotine groups, when compared to the placebo group (Pla-Nic1:t(52) = 2.734,p_corr_ = 0.009;Pla-Nic2:t(50) = 2.472,p_corr_ = 0.017). Hence, these group differences suggest that the effect of reduced CS-differentiation by nicotine during learning endures to later (drug-free) retrieval. Furthermore, acute administration of nicotine also reduces CS-differentiation during retrieval, even when the learning was unaffected by the drug (Fig. 2b). Additionally, our analysis revealed a main effect of acute nicotine administration on extinction of threat responses (F(2,124.48) = 4.465,p = 0.013), indicating trendwise increased fear ratings across both CSs in the group that received acute nicotine before extinction (Nic2), when compared to the placebo group (t(79) = 2.237,p_corr_ = 0.084).

##### US expectancy

We found a stimulus*group interaction (F(2,1219.09) = 4.656,p = 0.010, Fig. S1) at the end of EXT, but post-hoc comparisons of CS specific responses between groups revealed no differences. Furthermore, we found no group effect on US expectancy in the memory retrieval during the first block of EXT (F(2,1213.15) = 1.632,p = 0.196).

##### SCR

The analysis of SCRs revealed a stimulus*group interaction (F(2,717) = 4.922,p = 0.008, Fig. S3) in the last block of the EXT, which consisted of higher SCR towards the CS+ in the group of nicotine administration during acquisition (Nic1), when compared to the placebo group (Nic1–Pla:t(70) = 2.695,p_corr_ = 0.026; and compared to acute nicotine administration Nic1–Nic2:t(70) = 2.359,p_corr_ = 0.042). These results were against our hypothesis, but mirror the SCR results from day 1. We found no effects of nicotine administration on SCR during memory retrieval in the first block. The increase in CS-differentiation after nicotine administration might point towards a misalignment between enhanced psychophysiological arousal to threats together with a reduction in declarative identification of threat and safety (i.e., rating results).

##### fMRI

Next, we aimed to examine the effect of prior nicotine during acquisition (Nic1) and acute nicotine administration during extinction (Nic2) on hemodynamic responses during EXT (last block, as preregistered), both compared against placebo (Pla). Similar to our findings from ACQ, this analysis revealed that nicotine administration during acquisition of threat responses (Nic1), still led to reduced differential responses (CS + > CS-) in the left HC during the last block of EXT (T = 3.94,p_FWE_ = 0.010; Fig. 3c), when compared to the placebo group. This effect mirrors the effect of nicotine on patterns of hippocampal activity, as well as the fear ratings, during acquisition of threat responses. Additionally, we found that nicotine during ACQ reduced differential responses in extinction training within the bilateral INS (left INS:T = 4.60, p_FWE_ = 0.001;right INS:T = 3.82,p_FWE_ = 0.012) and right NAcc (T = 2.85,p_FWE_ = 0.036), when compared to the placebo group. The acute effect of nicotine on EXT was reflected by reduced differential responses in the left INS (T = 3.75,p_FWE_ = 0.021;Table S3/Fig. S4), as compared to placebo controls. Exploratory analysis of the first block of the EXT, indicated a trendwise reduced discrimination after acute nicotine administration (Nic2), as compared to placebo in the left NAcc (T = 2.67,p_FWE_ = 0.065). This effect mirrors the reduced discrimination in fear ratings in this group.

##### PPI

Similar to acquisition, we used functional connectivity analysis in the form of a PPI to investigate the effects of nicotine on the linkage between hippocampus actviation and other ROIs. Our PPI analyses, with the left hippocampus as the seed region, were computed for the last block of EXT. We found decreased functional connectivity between the left hippocampus and the vmPFC in the group that received nicotine before EXT (MNIxyz:-6,46,-8;T = 3.7, p_FWE_ = 0.045) compared to the placebo group (Fig. 3d).

## Discussion

Our study departed with the hypothesis that nicotine leads to decreased discrimination between danger and safety stimuli, when compared to the Placebo group. Our analyses of the fear ratings and fMRI results confirmed our hypotheses for the acquisition and partly for the extinction of learned threats. During Fear Acquisition, we found a decreased discrimination of rated fear in the group that received nicotine, when compared to the placebo group. Reflecting the fear ratings, the nicotine group also showed a lower differential (CS + > CS-) hippocampal activation than the placebo group. This effect of nicotine administration on Fear Acquisition was further extended to a decreased discrimination in subjective fear between the CS+ and CS- during memory retrieval (before EXT) on day 2. Furthermore, the deficit in threat and safety discrimination in hippocampal activity by nicotine administration during ACQ was still evident during the end of EXT, when compared to the placebo group. In striking similarity to these effects, acute nicotine administration before EXT also resulted in decreased differential fear ratings between the CS+ and CS-, accompanied by decreased differentiation in insular activity as well as reduced differential amygdala activation. The effect of nicotine on amygdala activation was most pronounced in the first block of acquisition, which is consistent with amygdala activation during early stages of fear conditioning in humans, with rapid habituation of responses to aversive stimuli [27]. The Return of Fear manipulation turned out to be relatively robust against nicotine effects over outcome measures.

Interestingly, although our results indicate reduced discrimination of threat and safety by nicotine administration on a declarative level and within key brain regions that underlie threat processing, we found enhanced psychophysiological arousal to the learned threat in the group that received nicotine during threat acquisition, as compared to placebo. In particular, the group that received nicotine on day 1 exhibited a larger CS-discrimination during the last block in both, ACQ and EXT than the placebo group. These results align with a previously reported effect of nicotine [28] and might indicate that nicotine enhances peripheral arousal to threats, while declarative threat assement (i.e., fear ratings) and their underlying neural systems are not clearly distinguished between threat and safety. This misalingment in the different outcome measures might point towards an altered salience processing by nicotinic mechanisms. Another possible explanation for the increased SCR in the nicotine group might be an activation of sweat glands, since nicotine increases general eccrine sweating [29]. However, this would have affected the SCR to both, the CS+ and the CS-, rather than the enhanced discrimination that is observed in our study. But regardless, both explanations support the effect of nicotine to impair declarative identification of threats, while the bodily arousal responses are enhanced.

Regarding self-reported fear of learned threats during extinction (day 2), we found reduced differential responses in both nicotine groups compared to placebo controls. The reduced responses in the group that received nicotine on day 1, are most likely due to impaired learning during acquisition, which is then evident in impaired retrieval on day 2. In the group that received nicotine before extinction, we found the most pronounced neural effect at the end of extinction, indicated by decreased insula activation and functional connectivity between the hippocampus and the vmPFC. This effect might underlie an impairment of the formation of extinction memory by nicotine [30, 31], which becomes more evident at end of learning. There is strong evidence from rodent and human studies that chronic nicotine disrupts discrimination of threat and safety learning [5, 14, 15]. Here we could show that already a single, small dose of acutely administered nicotine leads to similar changes in human learning processes. In addition to this effect of nicotine on memory formation, we found an immediate effect of nicotine administration on fear ratings during retrieval. Here, the salience coding within a network-loop that includes the hippocampus and the NAcc (see below), which is supported by the trend of reduced neural activity in the NAcc during the first extinction block, in the group that received nicotine compared to placebo.

Here we show that nicotine alters hippocampal processes that enable discrimination between threat and safety. Such a neural effect by nicotine in humans is not only in line with animal studies in which nicotine enhanced responses in the hippocampus (and amygdala) [7], but also with experiments that provide evidence for nicotinic effects on synaptic plasticity in the hippocampus [9, 10]. Both effects of nicotine, on the neural systems in general, as well as on their synaptic plasticity in particular, are likely to contribute to the nicotinic reduction in threat and safety processing, in line with the key role of the hippocampus to integrate learned threats and safety signals [32]. We further found that the functional connectivity between the hippocampus and the AMY and NAcc is enhanced by nicotine, which further aligns with our finding that both of these regions showed a similar decrement in discrimination of threat and safety signals during initial learning, as an effect of nicotine administration. Our results thereby imply that nicotine administration impairs differential activation to threat and safety cues within a network that is important for affective learning, including the hippocampus, SN/VTA, AMY and NAcc. Our findings thereby align with results in mice that show distinct activation of the VTA as a consequence of systemic nicotine administration that inhibits amygdala projections and increase anxiety-like behaviour [33].

The SN/VTA has previously been described to process new salient information within a network-loop that includes the hippocampus and the NAcc [34]. Furthermore, the SN/VTA in connection to the NAcc is part of the dopaminergic reward system, which is reinforced by nicotine intake [35]. In line with the aforementioned data in mice and the salience network-loop, we found that nicotine led to activation of the SN/VTA towards the CS+ , when participants expected the US, compared to placebo controls (importantly, the US expectancy did not differ between groups). The activation of the SN/VTA might be related to an amplification of a signal for a salient stimulus processing (CS+ when US is expected). The impaired discrimination between threat and safety in the hippocampus, NAcc and AMY could be one source that results in overactivation of the SN/VTA, by conveying enhanced salience (AMY) and novelty (hippocampus and NAcc [36]). Another possibility is that nicotine distorts SN/VTA processing by ramping up phasic signals to a salient stimulus (CS+ when a US is expected), which results in distorted hippocampal learning and salience integration in the AMY. Both possibilities highlight that nicotine distorts salience processing (encoded in the SN/VTA), which is related to impaired threat discrimination (in fear ratings and neural activation in the hippocampus, NAcc and AMY). These finding would align with the finer grained animal models of salience and novelty encoding along a VTA-hippocampal loop [34]. Our findings are further in line with findings in rodents that imply that NAcc dopamine signals saliency, even during aversive learning [36]. Nicotine modulates dopamine-transmission in the NAcc [37], which might have distorted salience processing in the NAcc that reduced the discrimination between CS+ and CS-.

A common result of these detailed mechanisms of nicotine during aversive learning would mean that the integration of salient new information about CS-US contingencies into long-term memory is distorted. Such an impairment in processing salient information with the SN/VTA, hippocampus, NAcc and AMY may then underlie impaired discrimination between threat and safety. Such a reduced discrimination is thought to reflect clinically relevant maladaptive aversive learning, as the same effect is found in patients with Anxiety disorders (AD) [38] and patients suffering from post-traumatic stress disorder (PTSD), showed decreased hippocampal activation during extinction recall, when compared to healthy controls [39]. Nicotine might effectively strengthen these deficits reported in patients with AD by distorting salience processing. The analysis of the US expectancy ratings, which rather reflects cognitive understanding of the paradigm, was not effected by nicotine on either day.

Our study contributes the effects of nicotine on aversive learning processes and adaptive switches to safety. A recent study found only weak behavioural effects of acute nicotine on cognitive stability and flexibility in non-smokers that were modulated in thalamic and parietal regions [40]. Additionally, acute nicotine in non-smokers was found to increase brain activity to unpleasant images in the amygdala, anterior cingulate cortex and basal ganglia [41]. In contrast, the reported effects here point towards altered learning mechanisms, since there was no group difference in aversiveness ratings towards the unpleasant electric stimulus that served as US.

The limitations of our study include that every participant regardless of their body weight received the same dose of nicotine. Additionally, we could only rely on self-reported non-smoking status, drug and psychiatric disorder history.

In conclusion, our study provided evidence how nicotine alters fear learning and extinction processes in humans. We found that nicotine impairs the discrimination between danger and safety. This is likely due to decreased differential activation in the hippocampus along with the amygdala and Ncl. Accumbens after nicotine administration. This study thereby reveals a mechanistic insight how nicotine administration leads to maladaptive aversive learning in humans and provides the neuropsychopharmacological link how smoking might contribute as a significant risk factor to the development and maintenance of pathological anxiety.

### Supplementary information

Supplement-Nicotine reduces discrimination between threat and safety in the hippocampus, nucleus accumbens and amygdala

## Data Availability

The rating and SCR data, as well as the contrast fMR images used in this study are available for download from: https://gin.g-node.org/MadeleineMueller/Mueller_et_al_2023_Nicotine_and_fear.
